# Association of Korean Healthy Eating Index with Phase Angle and Extracellular Water-to-Total Body Water Ratio: Findings from a Nationally Representative Study

**DOI:** 10.3390/nu18193213

**Published:** 2026-09-29

**Authors:** Seong-Uk Baek, Jin-Ha Yoon

**Affiliations:** 1 Graduate School, Yonsei University College of Medicine, Seoul 03722, Republic of Korea; 2 Department of Preventive Medicine, Yonsei University College of Medicine, Seoul 03722, Republic of Korea; 3 Institute for Innovation in Digital Healthcare, Yonsei University Health System, Seoul 03722, Republic of Korea; 4 The Institute for Occupational Health, Yonsei University College of Medicine, Seoul 03722, Republic of Korea

**Keywords:** bioimpedance analysis, body fluid, diet, nutrition

## Abstract

**Background:** Phase angle and the extracellular water-to-total body water (ECW/TBW) ratio, derived from bioelectrical impedance analysis, have attracted interest as biomarkers. This cross-sectional study analyzed the associations of dietary quality with phase angle and the ECW/TBW ratio. **Methods:** This study used a nationally representative sample of 12,162 adults from the Republic of Korea. Dietary quality was assessed using the Korean Healthy Eating Index (KHEI; range: 0–100), with higher scores indicating better dietary quality, and categorized into quartiles. Phase angle (°) and the ECW/TBW ratio (%) were measured using a bioelectrical impedance analyzer. Linear regression models were used to analyze associations between the KHEI and phase angle and the ECW/TBW ratio, with results reported as beta coefficients and 95% confidence intervals (CIs). **Results:** The mean (standard deviation) phase angle and ECW/TBW ratio were 5.37 (0.80)° and 38.19 (0.79)%, respectively. In the fully adjusted model, higher KHEI scores were associated with higher phase angle, with β (95% CI) values of 0.04 (0.01, 0.07), 0.06 (0.02, 0.09), and 0.08 (0.04, 0.11) for Q2, Q3, and Q4, respectively. Additionally, higher KHEI scores were associated with a lower ECW/TBW ratio, with β (95% CI) values of −0.03 (−0.07, −0.00), −0.06 (−0.09, −0.02), and −0.07 (−0.10, −0.03) for Q2, Q3, and Q4, respectively. **Conclusions:** Our findings showed that dietary quality was positively associated with phase angle and negatively associated with the ECW/TBW ratio. However, longitudinal studies are needed to confirm temporal relationships.

## 1. Introduction

Dietary quality is a multidimensional assessment of overall dietary intake and adherence to healthy eating patterns [1]. Higher dietary quality, often assessed using dietary indices including the Healthy Eating Index (HEI) or Mediterranean Diet Score, has been linked to a lower prevalence of cardiometabolic risk factors and reduced risk of cardiovascular events and all-cause mortality [2,3]. Additionally, poor dietary intake has been associated with greater exposure to environmental pollutants in the Republic of Korea [4,5,6]. A recent nationwide study showed in the Republic of Korea that overall dietary quality, as measured using the Korean Healthy Eating Index (KHEI), has steadily declined over the past decade [7]. This trend has been attributed to the increasing adoption of Westernized dietary patterns and the growing consumption of ultra-processed foods [7], underscoring the need for evidence-based policy interventions to improve dietary quality among the Korean population [8,9].

Bioelectrical impedance analysis (BIA) is a noninvasive and readily available method for assessing body composition and fluid distribution. Phase angle, which reflects the response of body tissues to an applied electrical current, is a recognized indicator of cellular membrane integrity [10,11]. The extracellular water-to-total body water (ECW/TBW) ratio may increase fluid retention or edema. Previous studies have shown that conditions such as systemic inflammation and sarcopenia are linked to lower phase angle and higher ECW/TBW ratio [12,13,14]. Although phase angle and the ECW/TBW ratio are distinct BIA-derived measures with different characteristics, they are closely related through shared physiological processes, including systemic inflammation, nutritional status, and fluid homeostasis [10,15]. Additionally, a low phase angle reflects reduced body cell mass and expansion of extracellular water, and phase angle has been shown to be strongly inversely correlated with the ECW/TBW ratio [16]. Furthermore, the aging process, which is characterized by physiological changes including frailty, sarcopenia, and chronic inflammation, may substantially affect phase angle and the ECW/TBW ratio [17,18]. Previous studies have documented that age serves as a key determinant of both phase angle and ECW/TBW ratio; older age has been associated with a lower phase angle and a higher ECW/TBW ratio [18,19]. Given that both measures can be assessed non-invasively using BIA, previous studies have used both measures as biomarkers of physiological status and as prognostic indicators of disease progression in epidemiological studies [13,20,21].

To date, few studies have examined the relationship between dietary quality and phase angle or the ECW/TBW ratio. The HEI was positively associated with phase angle among Brazilian adults [22]. Additionally, dietary quality was negatively associated with the ECW/TBW ratio in young Polish adults [23]. Higher dietary quality may be associated with lower oxidative stress and systemic inflammation [24], potentially supporting cellular membrane integrity and fluid homeostasis [11,13], which may in turn be reflected by a higher phase angle and a lower ECW/TBW ratio.

Despite existing evidence on the associations between dietary quality, phase angle, and the ECW/TBW ratio, several research gaps remain. In particular, population-level evidence examining the associations of overall dietary quality with both phase angle and the ECW/TBW ratio using nationally representative samples remains scarce in the literature. Additionally, dietary quality and eating practices vary substantially across cultural contexts. To the best of our knowledge, no previous study has analyzed the association between dietary quality and phase angle and the ECW/TBW ratio among Korean adults. Furthermore, phase angle and the ECW/TBW ratio reflect multiple physiological aspects, including fluid homeostasis, systemic inflammation, and body composition. Therefore, examining their associations with dietary quality may provide a more comprehensive understanding of the role of dietary quality in overall physiological status.

We hypothesized that (i) KHEI would be positively associated with phase angle and (ii) KHEI would be negatively associated with the ECW/TBW ratio. Therefore, this study aimed to examine the associations of dietary quality with phase angle and the ECW/TBW ratio among adults in the Republic of Korea.

## 2. Methods

### 2.1. Study Population

**Figure 1:**
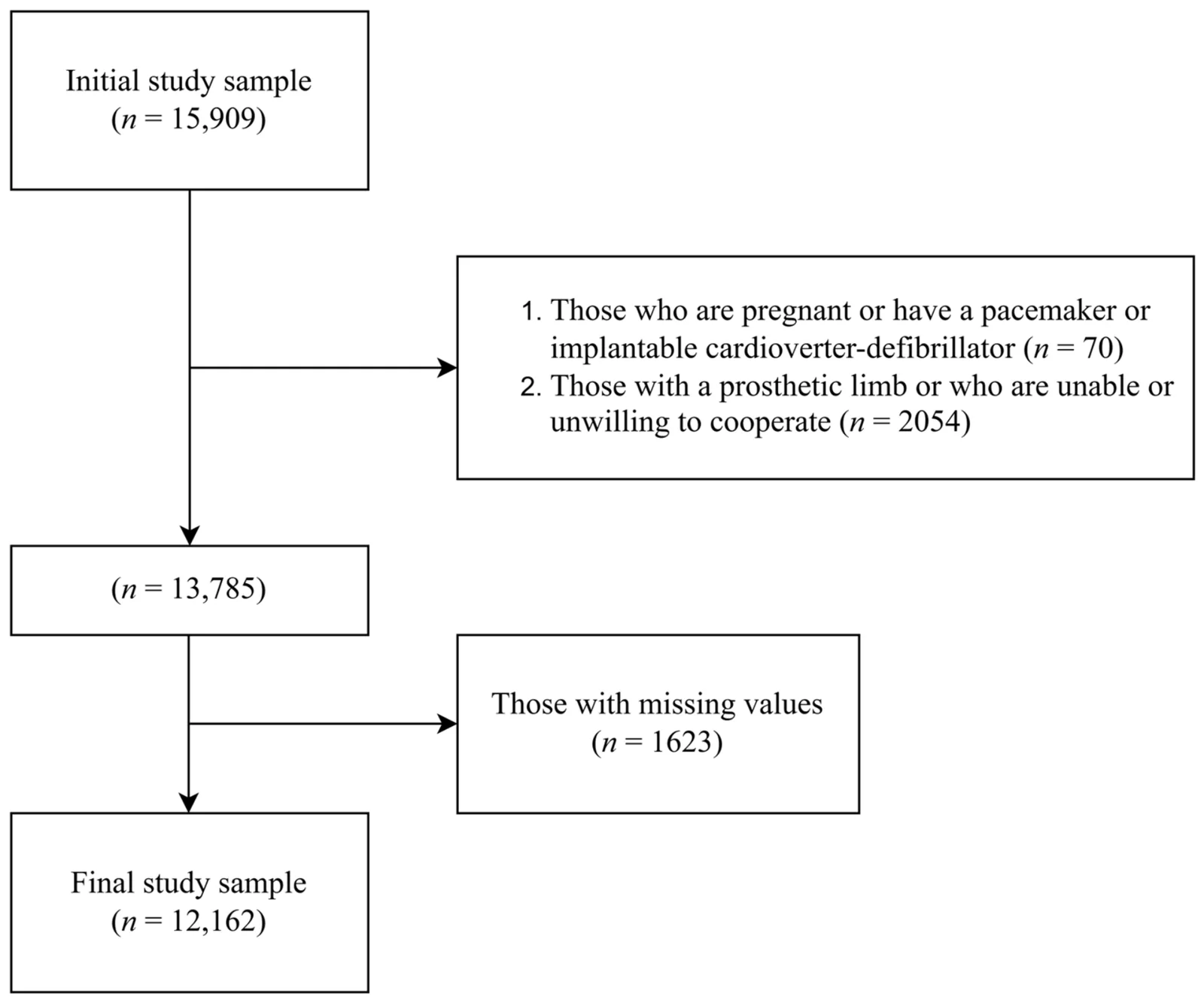
Study sample selection process.

Study participants were recruited from the Korea National Health and Nutrition Examination Survey (KNHANES). KNHANES employs a multistage, clustered, and systematic sampling procedure to recruit a nationally representative sample of the Korean population. As BIA was first incorporated in KNHANES in 2022, we included participants from the 2022–2024 KNHANES in the analysis. Figure 1 illustrates the sample selection flowchart. Initially, 15,909 individuals aged >18 years were included in the dataset. We excluded participants who were pregnant or had a pacemaker or implantable cardioverter-defibrillator (*n* = 70), as well as those with a prosthetic limb or who were unable or unwilling to cooperate (*n* = 2054). Finally, 12,162 participants with complete data on all study variables were included in the analysis after excluding 1623 individuals with missing data. The ethical approval was granted by the Institutional Review Board of the Korea Disease Control and Prevention Agency (2018-01-03-4C-A, 30 December 2021; 2022-11-16-R-A, 16 November 2022; 2022-11-16-R-03, 16 November 2022). All participants provided written informed consent.

### 2.2. KHEI

Dietary quality was evaluated using the KHEI, which comprises 14 items across three domains and yields a score ranging from 0 to 100, with higher scores indicating better dietary quality. Previous studies have demonstrated good reliability and validity of the KHEI [25]. The KHEI was calculated based on a 1-day 24 h dietary recall. Specifically, participants reported all foods consumed during the previous day with guidance and assistance from trained dietitians. Subsequently, food or nutrient intakes were determined using the Standard Food Composition Table (8th revision) [25]. The KHEI comprises three domains: an adequacy domain that assesses the intake of fruits, vegetables, and protein-rich foods; a moderation domain that evaluates the intake of saturated fatty acids, sodium, and sugars; and a balance domain that assesses the distribution of carbohydrates, fat, and total energy intake. Detailed survey items have been previously reported [25]. For the analysis, the KHEI was categorized into quartiles (Q1: <52.0; Q2: 52.0–61.2; Q3: 61.3–70.1; Q4: >70.1) and also analyzed as a continuous variable.

### 2.3. Phase Angle and ECW/TBW Ratio

Phase angle and the ECW/TBW ratio were measured using a multi-frequency bioelectrical impedance analyzer (InBody 970; InBody Co., Ltd., Seoul, Republic of Korea) with built-in software (version 970DM-0430; InBody Co., Ltd., Seoul, Republic of Korea). The InBody 970 has demonstrated good validity for body composition assessment [26]. In addition, measurements obtained using the InBody 970 have demonstrated high reliability compared with those from other BIA devices [27]. BIA was performed according to a standardized protocol. Participants fasted for at least 8 h, removed metal objects and electronic devices, and stood barefoot on the bioelectrical impedance analyzer while holding the hand electrodes. Whole-body phase angle was assessed using a 50 kHz frequency. Phase angle was calculated as arctan[reactance (Xc)/resistance (R)] × (180/π). Phase angle was recorded to the nearest 0.1°. ECW and TBW were measured to the nearest 0.1 L, and the ECW/TBW ratio was computed and expressed as a percentage (%).

### 2.4. Control Variables

The following sociodemographic and clinical characteristics were included in the analysis: sex, age, income (Q1–Q4), educational level (middle school or lower, high school, or college or higher), marital status (married or unmarried), smoking status (no or yes), physical activity (no or yes), alcohol use (Alcohol Use Disorder Identification Test-Consumption [AUDIT-C] score), body mass index (kg/m ^2^), estimated glomerular filtration rate (eGFR), diabetes (no or yes), and hypertension (no or yes). Physical activity was assessed using the Korean version of the Global Physical Activity Questionnaire and dichotomized based on the WHO physical activity guidelines as ≥150 min or <150 min of physical activity per week. eGFR was estimated using the European Kidney Function Consortium equation based on serum creatinine levels. Diabetes was defined as a fasting plasma glucose level ≥ 126 mg/dL, glycated hemoglobin ≥ 6.5%, or current use of insulin or oral glucose-lowering medication. Hypertension was defined as a resting blood pressure ≥ 140/90 mmHg or current use of oral antihypertensive medication.

### 2.5. Data Analysis

For descriptive analysis, sociodemographic characteristics were presented across KHEI quartiles. For regression analysis, multivariable linear regression models were used to examine the association between the KHEI and continuous phase angle and the ECW/TBW ratio values. Unadjusted, sex-, sex- and age-adjusted, and fully adjusted models were sequentially fitted to assess the potential influences of confounding. Beta coefficients (β) and 95% confidence intervals (CI) were estimated. The normal distributions of phase angle and the ECW/TBW ratio were confirmed by visual inspection of the histogram and Q–Q plots. Potential nonlinear associations between the KHEI and phase angle and the ECW/TBW ratio were examined using restricted cubic spline functions with four knots [28]. For sensitivity analysis, multiple imputation was performed using multivariate imputation by chained equations (MICE), generating 20 complete datasets. Data analyses were conducted using R software (version 4.6.0). A complex survey design was incorporated into the regression models using the survey package [29]. Following the KNHANES analysis guidelines, the sampling weights for each year were divided by three, corresponding to the number of survey cycles pooled, and then applied to the pooled 2022–2024 dataset [30]. The primary sampling units (PSUs) comprised 576 census blocks covering the entire country, with approximately 50–60 households included within each PSU. The strata were defined based on administrative region (si/do), urban/rural area (dong/eup/myeon), and housing type. The PSU and strata variables were specified as psu and kstrata, respectively, in the svydesign() function of the R survey package. Linear regression analysis was conducted using the svyglm function. Restricted cubic spline analyses were conducted using the rms package, and multiple imputations were performed using the mice package [31]. Restricted cubic spline functions were specified in the survey-weighted linear regression models using the rcs function from the R rms package. The resulting associations were visualized using the ggeffect function from the ggeffects package in R. The survey design was then incorporated into each imputed dataset using the original PSU, strata, and pooled sampling weight variables through the svydesign() function in the R survey package. Survey-weighted regression analyses were conducted separately across the 20 imputed datasets. Finally, the resulting estimates were then combined using MIcombine() in the R mitools package according to Rubin’s rules, with the pooled estimates and corresponding 95% CIs reported. Additionally, given the potential influence of reduced kidney disease on hydration status, a sensitivity analysis excluding participants with eGFR <60 mL/min/1.73 m^2^ was performed. Finally, the potential moderating role of age in the associations of KHEI with phase angle and the ECW/TBW ratio was explored by including an interaction term (age × KHEI continuous score) in each fully adjusted regression model. When a statistically significant interaction was observed, age-stratified analyses were conducted to examine age-specific associations of KHEI with phase angle and the ECW/TBW ratio. Participants were stratified into three age groups: young adults (19–39 years), middle-aged adults (40–59 years), and older adults (≥60 years).

## 3. Results

**Figure 2:**
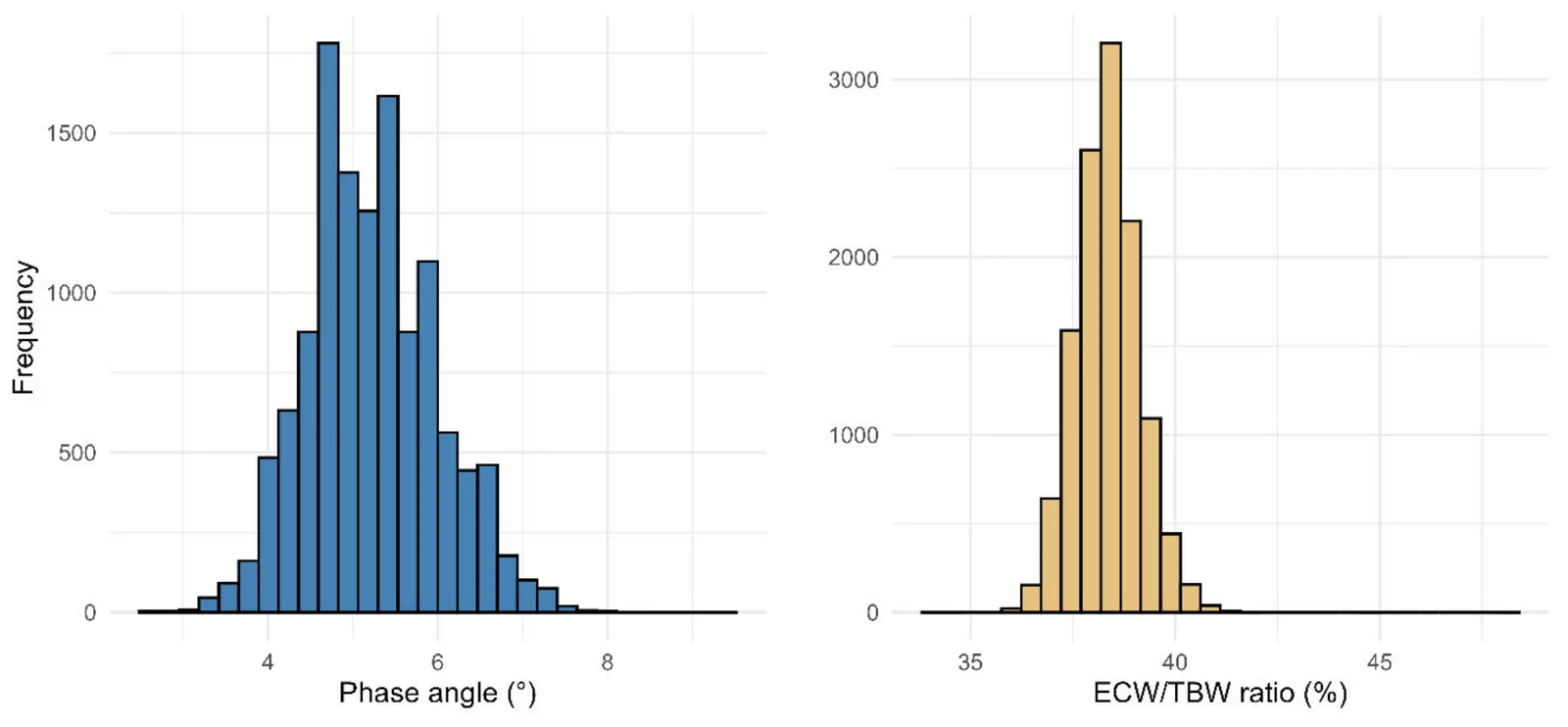
Distribution of phase angle and the ECW/TBW ratio (ECW/TBW, extracellular water-to- total body water ratio).

Table 1 presents participant characteristics according to KHEI categories. Compared with participants in the lowest KHEI category, those in the higher KHEI categories were more likely to be female, older, married, and nonsmokers, and to have higher income levels but lower educational levels. They also had lower AUDIT-C scores, body mass index, and eGFR, and were more likely to have diabetes mellitus and hypertension. Overall, the mean (standard deviation [SD]) phase angle was 5.37 (0.80)°, with values of 5.54 (0.80)°, 5.41 (0.81)°, 5.31 (0.77)°, and 5.15 (0.77)° across Q1–Q4 KHEI quartiles, respectively, showing a decreasing trend across higher KHEI quartiles. In contrast, the mean (SD) ECW/TBW ratio was 38.19 (0.79)%, with values of 37.97 (0.74)%, 38.16 (0.78)%, 38.27 (0.76)%, and 38.45 (0.78)% across Q1–Q4 KHEI quartiles, respectively, showing an increasing trend across higher KHEI quartiles. Figure 2 shows the distribution of phase angle and the ECW/TBW ratio in the overall sample.

Table 2 shows the relationship between the KHEI and phase angle. In the unadjusted model (Model 1), compared with the lowest KHEI quartile, higher KHEI quartiles were associated with lower phase angle, with adjusted β (95% CI) values of −0.13 (−0.18, −0.09), −0.23 (−0.28, −0.18), and −0.39 (−0.44, −0.34) for Q2, Q3, and Q4, respectively. These associations were attenuated, but remained negative after adjusting for sex (Model 2). However, after further adjustment for age (Model 3), the direction of the associations was reversed, with β (95% CI) values of 0.03 (0.00, 0.07), 0.04 (0.00, 0.07), and 0.04 (0.00, 0.07) for Q2, Q3, and Q4, respectively. In the fully adjusted model (Model 4), higher KHEI scores were consistently associated with higher phase angle, with β (95% CI) values of 0.04 (0.01, 0.07), 0.06 (0.02, 0.09), and 0.08 (0.04, 0.11) for Q2, Q3, and Q4, respectively. Similarly, a 1-SD increment in KHEI score was associated with a 0.03° higher phase angle (95% CI, 0.02 to 0.04).

Table 3 presents the associations between the KHEI and the ECW/TBW ratio. In the unadjusted model (Model 1), higher KHEI scores were associated with a higher ECW/TBW ratio, with β (95% CI) values of 0.19 (0.15, 0.24), 0.30 (0.25, 0.34), and 0.48 (0.44, 0.53) for Q2, Q3, and Q4, respectively, compared with Q1. These associations were attenuated but remained positive after adjusting for sex (Model 2). However, after further adjustment for age (Model 3), the direction of the associations was reversed, with β (95% CI) values of −0.04 (−0.07, −0.01), −0.06 (−0.10, −0.03), and −0.07 (−0.10, −0.03) for Q2, Q3, and Q4, respectively. The inverse associations remained consistent in the fully adjusted model (Model 4), with β (95% CI) values of −0.03 (−0.07, −0.00), −0.06 (−0.09, −0.02), and −0.07 (−0.10, −0.03) for Q2, Q3, and Q4, respectively. Similarly, a 1-SD increment in KHEI score was associated with a 0.03 percentage-point lower ECW/TBW ratio (95% CI, −0.04 to −0.02).

**Figure 3:**
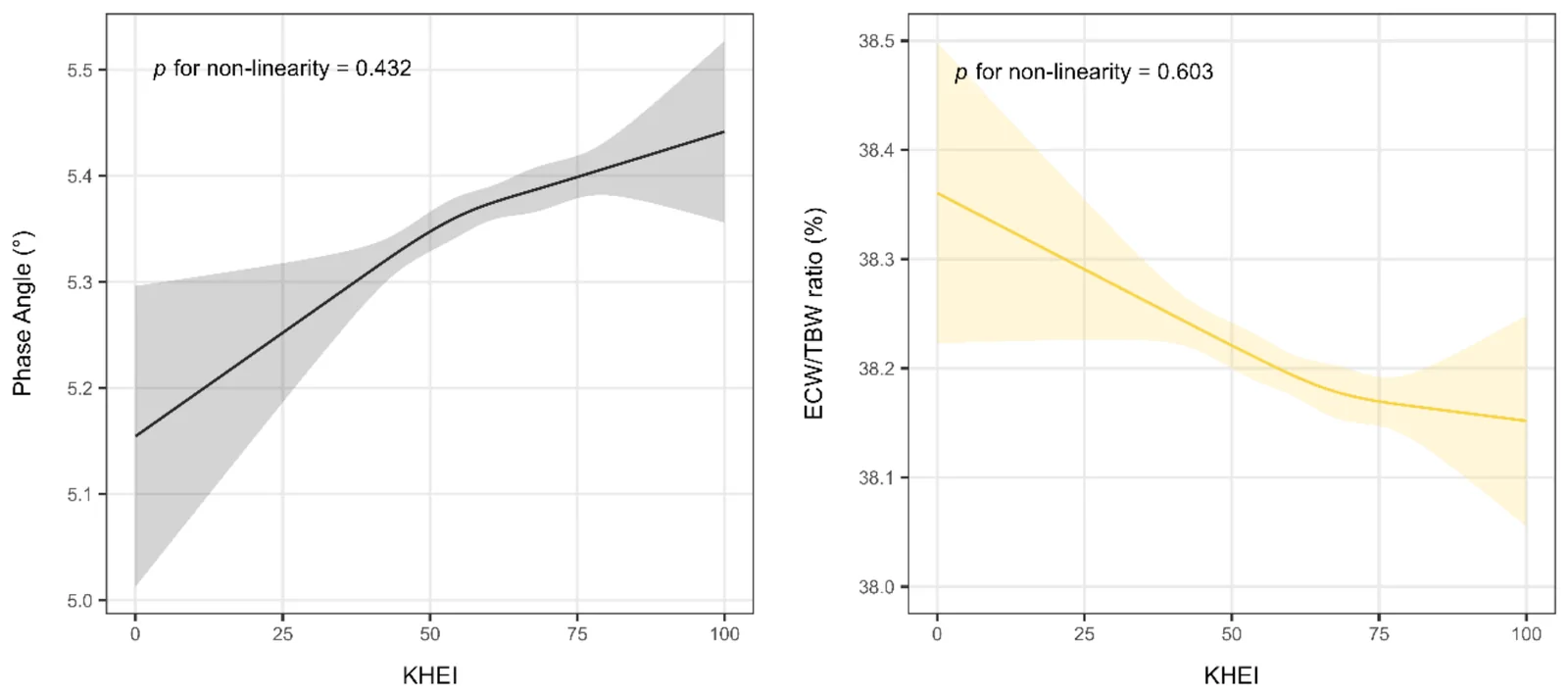
Nonlinear associations of the KHEI with phase angle and the ECW/TBW ratio using restricted cubic spline functions. The models were adjusted for sex, age, income, education, marital status, smoking, physical activity, alcohol use, body mass index, estimated glomerular filtration rate, diabetes mellitus, and hypertension. (KHEI, Korean Healthy Eating Index; ECW/TBW, extracellular water-to-total body water ratio).

Figure 3 presents the results of the nonlinear models. As the KHEI increased, the predicted phase angle increased, whereas the predicted ECW/TBW ratio decreased. The *p*-values for nonlinearity were 0.432 and 0.603 for the phase angle and ECW/TBW ratio models, respectively, indicating no clear evidence of nonlinear associations between the KHEI and either outcome.

The results of the sensitivity analysis using multiple imputations are presented in Table S1. These results are largely consistent with those of the primary analyses. In the fully adjusted model, compared with the lowest KHEI quartile, adjusted β (95% CI) values for phase angle were 0.04 (0.01, 0.07), 0.06 (0.02, 0.09), and 0.08 (0.04, 0.11) for Q2, Q3, and Q4 KHEI quartiles, respectively. Similarly, compared with the lowest KHEI quartile, adjusted β (95% CI) values for the ECW/TBW ratio were −0.03 (−0.07, −0.00), −0.06 (−0.09, −0.02), and −0.07 (−0.10, −0.03) for Q2, Q3, and Q4 KHEI quartiles, respectively. A sensitivity analysis excluding participants with eGFR <60 mL/min/1.73 m^2^ yielded similar results for the associations of the KHEI with both phase angle and ECW/TBW (Table S2). Finally, the interaction terms between age and KHEI were statistically significant for both phase angle and the ECW/TBW ratio (Table S3). In age-stratified analyses (19–39, 40–59, and ≥60 years), the positive association between KHEI and phase angle was more pronounced among older adults. Similarly, the negative association between KHEI and the ECW/TBW ratio was more pronounced among older adults, whereas no clear associations were observed among young adults for either outcome (Table 4).

## 4. Discussion

The present study examined the association of dietary quality, as measured by the KHEI, with phase angle and the ECW/TBW ratio. In the crude models, a higher KHEI score was associated with a lower phase angle and a higher ECW/TBW ratio; however, after adjusting for age and other sociodemographic characteristics, the direction of the associations was reversed, with a higher KHEI score associated with a higher phase angle and a lower ECW/TBW ratio. However, it should be noted that statistically significant associations do not necessarily translate into clinically meaningful differences in magnitude. Given the modest effect sizes and the cross-sectional nature of the study design, the findings should be interpreted with caution. For instance, the adjusted beta coefficients for the KHEI Q4 category, with Q1 as the reference category, were 0.08° for phase angle and −0.07% for the ECW/TBW ratio. Although no universal guidelines have established thresholds for clinically meaningful differences at the individual level, these magnitudes were substantially smaller than those reported as clinically meaningful in previous studies, which suggested thresholds of approximately 0.9–1.0° for phase angle and 1% for the ECW/TBW ratio [19,32]. However, no established threshold has been defined for a clinically meaningful difference in phase angle or the ECW/TBW ratio. Nevertheless, this study is among the first to report the relationship between the KHEI and phase angle and the ECW/TBW ratio among adults in the Republic of Korea.

Unexpectedly, participants in higher KHEI quartiles had a lower phase angle and a higher ECW/TBW ratio in the descriptive analysis; however, after adjustment for age, the direction of these associations was reversed. These observations suggest that age may be an important confounder of the associations between dietary quality and phase angle and the ECW/TBW ratio. This is consistent with prior findings that overall dietary quality tends to improve with age [7], while older adults have substantially lower phase angles and higher ECW/TBW ratios [18,33]. Furthermore, our analysis showed a significant moderating role of age in the associations of KHEI with both phase angle and the ECW/TBW ratio. In age-stratified analyses, the associations of KHEI with both phase angle and the ECW/TBW ratio were more pronounced among adults aged ≥60 years.

Previous studies on the relationship between dietary quality and phase angle or the ECW/TBW ratio have reported inconsistent results. For instance, a Brazilian study reported that higher dietary quality, measured using the HEI, was associated with lower odds of having a low phase angle [22]. A study of 98 individuals undergoing hemodialysis showed that poor nutritional status, as assessed by Nutrition Risk Score and Malnutrition Inflammation Score, was associated with a lower phase angle [34]. In contrast, a study of Iranian adults found no meaningful association between dietary quality, as measured by the dietary inflammatory index, and phase angle [35]. These discrepant findings may be attributable to several factors, including the relatively modest sample sizes of previous studies and differences in the measures used to assess dietary quality across studies. Evidence regarding the association between dietary quality and the ECW/TBW ratio remains limited. Nevertheless, an inverse association between dietary quality and the extracellular mass-to-body cell mass ratio has been reported [36], which is consistent with our findings.

The precise mechanisms underlying the associations between favorable dietary quality, higher phase angle, and lower ECW/TBW ratio are not fully understood. One potential biological pathway may involve the mitigation of oxidative stress and systemic inflammation. Individuals with higher KHEI scores may have higher intakes of antioxidants, which may be associated with lower levels of chronic low-grade inflammation [37]. Favorable dietary quality, as measured by the HEI, was inversely associated with circulating biomarkers of inflammation, including C-reactive protein and interleukin-6 [38,39]. Chronic inflammation may promote reactive oxygen species production and cellular injury [11]. Additionally, chronic inflammation may increase vascular permeability and facilitate fluid leakage into the interstitial space, which may contribute to increased ECW [40]. Thus, favorable dietary quality may be associated with a higher phase angle and lower ECW/TBW ratio through pathways involving reduced chronic inflammation. Previous studies have demonstrated that muscle quality and mass are positively associated with phase angle and negatively associated with the ECW/TBW ratio [16,41]. Favorable dietary quality may be associated with a higher phase angle and lower ECW/TBW ratio by improving body composition through the consumption of protein-rich and other nutrient-dense foods [42,43].

This study has some limitations. First, the causal relationship between dietary quality, phase angle, and the ECW/TBW ratio could not be established due to the cross-sectional nature of the study. Additionally, the possibility of reverse causality should be considered, whereby individuals with chronic conditions may modify their dietary behaviors following diagnosis. Future longitudinal studies should verify the temporal ordering between the KHEI and BIA-derived measures. Second, the KHEI was assessed using the 24 h recall method, which may not accurately reflect an individual’s usual long-term dietary quality. Previous studies have noted that the 24 h dietary recall method is susceptible to random measurement errors, which may reduce the precision of dietary quality assessment [44,45]. In addition, recall bias may be more pronounced among individuals with chronic conditions or impaired cognitive function. Social desirability bias may also lead some individuals to under-report their energy and food intake [46]. Such measurement errors may bias the observed associations; however, the direction and magnitude of this bias cannot be determined from the available data. Future studies should repeatedly assess dietary quality over time to enhance measurement accuracy. In addition, although dietary quality was assessed by trained dietitians, the assessment method was subject to recall bias. Third, the participants were limited to community-dwelling adults in the Republic of Korea. Given cultural differences in dietary quality, practices, and body composition, the findings of this study may not be generalizable to populations in other countries or cultural settings. Fourth, the potential for residual confounding should be noted. For instance, while we adjusted for kidney function using eGFR, information on heart failure, liver disease, diuretic use, and other conditions that may affect hydration status was not available in the dataset. These unmeasured factors might have biased the observed associations between dietary quality and BIA-derived outcomes.

Nevertheless, this study provides novel evidence on the associations of dietary quality with phase angle and the ECW/TBW ratio, which have rarely been investigated in existing literature. Furthermore, the use of a nationally representative sample enhances the generalizability and robustness of the findings.

## 5. Conclusions

This study analyzed the association between dietary quality and phase angle and the ECW/TBW ratio in adults in the Republic of Korea. After adjusting for sociodemographic and health-related characteristics, dietary quality exhibited a positive association with phase angle and a negative association with the ECW/TBW ratio. Given the modest effect sizes and the cross-sectional nature of the study design, the findings should be interpreted with caution.

## Figures and Tables

**Table 1:** Participant characteristics according to KHEI quartiles.

	Overall	KHEI Quartiles			
		Q1 (Lowest)	Q2	Q3	Q4 (Highest)
	*n* = 12,162	*n* = 3060	*n* = 2980	*n* = 3041	*n* = 3081
Sex					
Male	5235 (49.53%)	1411 (53.62%)	1351 (51.36%)	1324 (48.82%)	1149 (42.70%)
Female	6927 (50.47%)	1649 (46.38%)	1629 (48.64%)	1717 (51.18%)	1932 (57.30%)
Age					
Mean (SD)	51.01 (16.01)	42.19 (14.79)	50.27 (15.31)	54.36 (14.84)	60.10 (13.04)
Income					
Q1 (lowest)	2910 (23.39%)	884 (27.71%)	729 (23.41%)	711 (22.43%)	586 (18.58%)
Q2	3064 (25.10%)	781 (25.77%)	787 (26.12%)	755 (24.68%)	741 (23.46%)
Q3	3098 (25.71%)	726 (24.80%)	747 (25.39%)	781 (25.67%)	844 (27.36%)
Q4 (highest)	3090 (25.80%)	669 (21.72%)	717 (25.09%)	794 (27.21%)	910 (30.60%)
Educational level					
Middle school or lower	3299 (19.96%)	510 (11.22%)	779 (19.33%)	888 (22.18%)	1122 (30.06%)
High school	4052 (34.71%)	1136 (38.59%)	969 (34.46%)	998 (33.85%)	949 (30.70%)
College or above	4811 (45.33%)	1414 (50.19%)	1232 (46.21%)	1155 (43.97%)	1010 (39.24%)
Marital status					
Married	8474 (66.68%)	1729 (53.37%)	2066 (66.73%)	2291 (73.56%)	2388 (77.12%)
Unmarried or others	3688 (33.32%)	1331 (46.63%)	914 (33.27%)	750 (26.44%)	693 (22.88%)
Smoking					
No	10,357 (82.72%)	2356 (74.67%)	2479 (81.23%)	2646 (85.48%)	2876 (92.29%)
Yes	1805 (17.28%)	704 (25.33%)	501 (18.77%)	395 (14.52%)	205 (7.71%)
Physical activity					
No	6743 (52.31%)	1648 (50.63%)	1665 (52.78%)	1685 (52.74%)	1745 (53.55%)
Yes	5419 (47.69%	1412 (49.37%)	1315 (47.22%)	1356 (47.26%)	1336 (46.45%)
AUDIT-C					
Mean (SD)	3.75 (3.61)	4.79 (3.74)	3.90 (3.60)	3.35 (3.46)	2.59 (3.17)
Body mass index (kg/m^2^)					
Mean (SD)	24.21 (3.77)	24.39 (4.17)	24.35 (3.76)	24.15 (3.59)	23.85 (3.36)
eGFR					
Mean (SD)	90.72 (15.90)	96.36 (14.76)	91.48 (15.60)	89.03 (15.42)	84.06 (15.39)
Diabetes mellitus					
No	10,217 (86.16%)	2750 (91.22%)	2528 (87.10%)	2492 (83.83%)	2447 (80.81%)
Yes	1945 (13.84%)	310 (8.78%)	452 (12.90%)	549 (16.17%)	634 (19.19%)
Hypertension					
No	7864 (69.31%)	2285 (78.26%)	1952 (70.09%)	1897 (66.54%)	1730 (59.33%)
Yes	4298 (30.69%)	775 (21.74%)	1028 (29.91%)	1144 (33.46%)	1351 (40.67%)
Phase angle (°)					
Mean (SD)	5.37 (0.80)	5.54 (0.80)	5.41 (0.81)	5.31 (0.77)	5.15 (0.77)
ECW/TBW ratio (%)					
Mean (SD)	38.19 (0.79)	37.97 (0.74)	38.16 (0.78)	38.27 (0.76)	38.45 (0.78)

AUDIT-C, Alcohol Use Disorder Identification Test-Consumption; KHEI, Korean Healthy Eating Index; SD, standard deviation; eGFR, estimated glomerular filtration rate; ECW/TBW ratio, extracellular water-to-total body water ratio. Values are presented as unweighted *n* with survey-weighted percentages or as survey-weighted means with standard deviations.

**Table 2:** Association between KHEI and phase angle using multivariable linear regression models.

	Model 1	Model 2	Model 3	Model 4
	β (95% CI)	β (95% CI)	β (95% CI)	β (95% CI)
**KHEI quartiles**				
Q1 (lowest)	Reference	Reference	Reference	Reference
Q2	−0.13 (−0.18, −0.09)	−0.11 (−0.14, −0.08)	0.03 (0.00, 0.07)	0.04 (0.01, 0.07)
Q3	−0.23 (−0.28, −0.18)	−0.18 (−0.22, −0.14)	0.04 (0.00, 0.07)	0.06 (0.02, 0.09)
Q4 (highest)	−0.39 (−0.44, −0.34)	−0.28 (−0.32, −0.24)	0.04 (0.00, 0.07)	0.08 (0.04, 0.11)
**KHEI score**				
1-SD increment ^a^	−0.14 (−0.16, −0.13)	−0.11 (−0.12, −0.09)	0.02 (0.01, 0.03)	0.03 (0.02, 0.04)

KHEI, Korean Healthy Eating Index; CI, confidence interval; SD, standard deviation. ^a^ 1-SD = 13.1. Model 1, unadjusted model; Model 2, adjusted for sex; Model 3, adjusted for sex and age; Model 4, adjusted for sex, age, income, education, marital status, smoking, physical activity, alcohol use, body mass index, estimated glomerular filtration rate, diabetes mellitus, and hypertension.

**Table 3:** Association between KHEI and the ECW/TBW ratio using multivariable linear regression models.

	Model 1	Model 2	Model 3	Model 4
	β (95% CI)	β (95% CI)	β (95% CI)	β (95% CI)
**KHEI quartiles**				
Q1 (lowest)	Reference	Reference	Reference	Reference
Q2	0.19 (0.15, 0.24)	0.18 (0.14, 0.22)	−0.04 (−0.07, −0.01)	−0.03 (−0.07, −0.00)
Q3	0.30 (0.25, 0.34)	0.27 (0.23, 0.31)	−0.06 (−0.10, −0.03)	−0.06 (−0.09, −0.02)
Q4 (highest)	0.48 (0.44, 0.53)	0.42 (0.37, 0.46)	−0.07 (−0.10, −0.03)	−0.07 (−0.10, −0.03)
**KHEI score**				
1-SD increment ^a^	0.18 (0.17, 0.20)	0.16 (0.15, 0.17)	−0.03 (−0.04, −0.02)	−0.03 (−0.04, −0.02)

KHEI, Korean Healthy Eating Index; CI, confidence interval; SD, standard deviation. ^a^ 1-SD = 13.1. Model 1, unadjusted model; Model 2, adjusted for sex; Model 3, adjusted for sex and age; Model 4, adjusted for sex, age, income, education, marital status, smoking, physical activity, alcohol use, body mass index, estimated glomerular filtration rate, diabetes mellitus, and hypertension.

**Table 4:** Association between KHEI and phase angle and ECW/TBW ratio using multivariable linear regression models. Stratified analysis by age groups.

	Phase Angle	ECW/TBW Ratio
	β (95% CI)	β (95% CI)
**Age = 19–39 years (young adults)**		
**KHEI quartiles**		
Q1 (lowest)	Reference	Reference
Q2	0.00 (−0.06, 0.06)	0.02 (−0.04, 0.08)
Q3	−0.02 (−0.09, 0.05)	0.03 (−0.04, 0.11)
Q4 (highest)	0.02 (−0.07, 0.11)	0.03 (−0.07, 0.13)
**KHEI score**		
1-SD increment ^a^	0.00 (−0.02, 0.03)	0.01 (−0.01, 0.04)
**Age = 40–59 years (middle-aged adults)**		
**KHEI quartiles**		
Q1 (lowest)	Reference	Reference
Q2	0.04 (−0.01, 0.08)	−0.05 (−0.10, −0.00)
Q3	0.04 (−0.01, 0.08)	−0.04 (−0.09, 0.01)
Q4 (highest)	0.06 (0.01, 0.11)	−0.03 (−0.08, 0.03)
**KHEI score**		
1-SD increment ^a^	0.03 (0.01, 0.05)	−0.02 (−0.03, 0.00)
**Age ≥ 60 years (older adults)**		
**KHEI quartiles**		
Q1 (lowest)	Reference	Reference
Q2	0.05 (0.00, 0.11)	−0.03 (−0.10, 0.03)
Q3	0.11 (0.06, 0.16)	−0.12 (−0.17, −0.06)
Q4 (highest)	0.11 (0.06, 0.16)	−0.13 (−0.18, −0.07)
**KHEI score**		
1-SD increment ^a^	0.04 (0.02, 0.06)	−0.05 (−0.07, −0.03)

KHEI, Korean Healthy Eating Index; CI, confidence interval; SD, standard deviation. ^a^ 1-SD = 13.1. All models adjusted for sex, age, income, education, marital status, smoking, physical activity, alcohol use, body mass index, estimated glomerular filtration rate, diabetes mellitus, and hypertension.

## Data Availability

Dataset can be obtained at https://knhanes.kdca.go.kr (accessed on 3 June 2026).

## Supplementary Materials

The following supporting information can be downloaded at https://www.mdpi.com/article/10.3390/nu18193213/s1, Table S1 Associations of the KHEI with phase angle and the ECW/TBW ratio using multivariable linear regression models. Sensitivity analysis using multiple imputation (n = 15,909); Table S2 Associations of the KHEI with phase angle and the ECW/TBW ratio using multivariable linear regression models. Sensitivity analysis excluding those with eGFR <60 mL/min/1.73 m^2^; Table S3 Association between KHEI and phase angle or ECW/TBW ratios using multivariable linear regression models with an interaction term between KHEI and age.
