# Supplementary material for: Association of Korean Healthy Eating Index with Phase Angle and Extracellular Water-to-Total Body Water Ratio: Findings from a Nationally Representative Study
Source: Nutrients. 2026 Sep 29;18(19):3213. doi: 10.3390/nu18193213 (PMC13667239; doi:10.3390/nu18193213)
Supplement: Supplementary file 1 [file nutrients-18-03213-s001.zip › nutrients-4586831-supplementary.pdf]

Table S1 Associations of the KHEI with phase angle and the ECW/TBW ratio using multivariable linear regression models. Sensitivity analysis using multiple imputation (n = 15,909)

	**Model 1**	**Model2**
	**Phase angle**	**ECW/TBW ratio**
	**β (95% CI)**	**β (95% CI)**
**KHEI quartiles**		
Q1 (lowest)	Reference	Reference
Q2	0.04 (0.01, 0.07)	−0.03 (−0.07, −0.00)
Q3	0.06 (0.02, 0.09)	−0.06 (−0.09, −0.02)
Q4 (highest)	0.08 (0.04, 0.11)	−0.07 (−0.10, −0.03)
**KHEI score**		
1-SD increment[a]	0.03 (0.02, 0.05)	−0.03 (−0.05, −0.02)

KHEI, Korean Healthy Eating Index; ECW/TBW, extracellular water-to-total body water ratio; CI, confidence interval; SD, standard deviation

[a]1-SD = 13.1

All models were adjusted for sex, age, income, education, marital status, smoking, physical activity, alcohol use, body mass index, estimated glomerular filtration rate, diabetes mellitus, and hypertension

Table S2 Associations of the KHEI with phase angle and the ECW/TBW ratio using multivariable linear regression models. Sensitivity analysis excluding those with eGFR <60 mL/min/1.73 m²

	**Model 1**	**Model2**
	**Phase angle**	**ECW/TBW ratio**
	**β (95% CI)**	**β (95% CI)**
**KHEI quartiles**		
Q1 (lowest)	Reference	Reference
Q2	0.04 (0.01, 0.07)	−0.03 (−0.07, −0.00)
Q3	0.05 (0.02, 0.08)	−0.05 (−0.08, −0.02)
Q4 (highest)	0.07 (0.04, 0.11)	−0.06 (−0.10, −0.03)
**KHEI score**		
1-SD increment[a]	0.03 (0.02, 0.04)	−0.03 (−0.04, −0.01)

KHEI, Korean Healthy Eating Index; ECW/TBW, extracellular water-to-total body water ratio; CI, confidence interval; SD, standard deviation

[a]1-SD = 13.1

All models were adjusted for sex, age, income, education, marital status, smoking, physical activity, alcohol use, body mass index, estimated glomerular filtration rate, diabetes mellitus, and hypertension

Table S3 Association between KHEI and phase angle or ECW/TBW ratios using multivariable linear regression models with an interaction term between KHEI and age

	**Phase angle**	**ECW/TBW ratio**
	**β (95% CI)**	**β (95% CI)**
**KHEI score**		
1-SD increment[a]	0.153 (0.113, 0.194)	–0.107 (–0.148, –0.066)
**KHEI score × age**	–0.002 (–0.003, –0.002)[b]	0.002 (0.001, 0.002)[b]

KHEI, Korean Healthy Eating Index; CI, confidence interval; SD, standard deviation
[a]1-SD = 13.1; [b] $p < 0.001$
All models adjusted for sex, age, income, education, marital status, smoking, physical activity, alcohol use, body mass index, estimated glomerular filtration rate, diabetes mellitus, and hypertension.
